# Effectiveness of influenza vaccination in preventing influenza in primary care, Navarre, Spain, 2021/22

**DOI:** 10.2807/1560-7917.ES.2022.27.26.2200488

**Published:** 2022-06-30

**Authors:** Iván Martínez-Baz, Itziar Casado, Ana Miqueleiz, Ana Navascués, Francisco Pozo, Camino Trobajo-Sanmartín, Esther Albéniz, Fernando Elía, Cristina Burgui, Miguel Fernández-Huerta, Carmen Ezpeleta, Jesús Castilla

**Affiliations:** 1 Instituto de Salud Pública de Navarra, Pamplona, Spain; 2 CIBER Epidemiología y Salud Pública (CIBERESP), Spain; 3 Navarre Institute for Health Research (IdiSNA), Pamplona, Spain; 4 Clinical Microbiology Department, Hospital Universitario de Navarra, Pamplona, Spain; 5 National Centre for Microbiology, Instituto de Salud Carlos III, Madrid, Spain; 6 Gerencia de Atención Primaria, Servicio Navarro de Salud-Osasunbidea, Pamplona, Spain

**Keywords:** influenza, influenza vaccine, acute respiratory infection, case-control study, vaccine effectiveness, repeated vaccination

## Abstract

Compared with individuals unvaccinated in the current and three previous influenza seasons, in 2021/22, influenza vaccine effectiveness at primary care level was 37% (95% CI: 16 to 52) for current season vaccination, regardless of previous doses, and 35% (95% CI: −3 to 45) for only previous seasons vaccination. Against influenza A(H3N2), estimates were 39% (95% CI: 16 to 55) and 24% (95% CI: −8 to 47) suggesting moderate effectiveness of current season vaccination and possible remaining effect of prior vaccinations.

The influenza season 2021/22 in Spain and Europe was characterised by low incidence of cases, long duration, predominance of influenza A(H3N2), and sporadic circulation of influenza A(H1N1)pdm09 and B/Victoria viruses [1,2]. While interim estimates from Denmark and the United States showed a low influenza vaccine effectiveness (IVE) against influenza A(H3N2) virus at primary care level [3,4], the end of season IVE still needs to be determined. It also remains to be established if vaccination in previous influenza seasons modified the IVE.

We aimed to estimate the effectiveness of the influenza vaccinations received in the current and previous seasons in preventing laboratory-confirmed influenza among patients attending primary care in the 2021/22 season. These data may contribute to upcoming decisions for the 2023 vaccine strain selection for the southern hemisphere.

## Setting and information sources

We performed a test-negative case–control study out in primary care in the Navarre region in northern Spain. In October and November 2021, the inactivated influenza vaccine was offered free of charge to all people aged 60 years or older and those aged 6 months or over with major chronic conditions. The trivalent adjuvanted vaccine (Chiromas, Sequirus, Siena, Italy) was mainly used in people older than 65 years and the tetravalent unadjuvanted vaccine (Vaxigrip Tetra, Sanofi Pasteur, Lyon, France) in the younger population.

Influenza vaccination status in the current and three previous seasons (2018/19 to 2021/22) was obtained from the online regional vaccination register [5]. Individuals were considered to be protected 14 days after vaccine administration.

From all acute respiratory infection (ARI) cases detected in primary care, nasopharyngeal and pharyngeal swabs were collected. Samples from patients who started symptoms in the previous 5 days were tested by antigen rapid test for severe acute respiratory syndrome coronavirus 2 (SARS-CoV-2) that causes coronavirus 19 disease (COVID-19). Symptomatic patients with a negative antigen test result for SARS-CoV-2, or those who consulted more than 5 days after symptom onset, were tested by RT-PCR assay for SARS-CoV-2 and influenza viruses. Whole genome sequencing or partial sequencing by Sanger was done in some strains of each week. A phylogenetic analysis was performed using the HA1 subunit of the haemagglutinin gene.

## Statistical analysis

The study population included ARI patients covered by the Navarre Health Service who were seen in primary care and tested for influenza virus from 22 November 2021 to 22 May 2022. We compared the vaccination status of laboratory-confirmed influenza patients (cases) and those who tested negative for influenza (controls). Nursing home residents and children younger than 9 years were excluded from the present study, as well as confirmed COVID-19 cases from the control group. However, patients with influenza and COVID-19 coinfection were included in the study.

Crude and adjusted odds ratios (OR) with their 95% confidence intervals (CI) were calculated using logistic regression. Adjusted models included sex, age group (9–24, 25–44, 45–64, 65–84 and ≥ 85 years), major chronic conditions, and month of swabbing. Although we performed the classical analysis including only current season vaccination, we considered as final IVE estimates those from models that included vaccination status in three previous seasons, using individuals unvaccinated in all seasons as reference category [6]. IVE was estimated as a percentage: (1–OR) x 100. Stratified analyses were performed by period (November–January and February–May), age groups (≤ 64 and ≥ 65 years) and in the target population.

A sensitivity analysis was performed including COVID-19 confirmed cases in the control group.

## Vaccination effectiveness against laboratory-confirmed influenza

Among 10,655 ARI patients included, 644 (6%) were confirmed influenza cases and 20 of them were also confirmed COVID-19 cases. Influenza cases were detected over a long period (7 months) with peaks in December and March (Table 1). Among influenza cases, 513 were due to A(H3N2), 2 to A(H1N1), 1 to B, and 128 to A non-subtyped virus. Cases were compared with 10,011 control patients with a negative result for any influenza virus.

**Table 1:** Characteristics of the patients with acute respiratory infection included in the test negative case–control analysis, Navarre, Spain, 22 November 2021–22 May 2022 (n = 10,655)

Variables	Laboratory-confirmed influenza cases n = 644		Influenza negative controls n = 10,011		p value
	n	%	n	%	
**Age groups (years)**					
9–24	213	33	1,674	17	< 0.001
25–44	193	30	3,292	33	
45–64	132	21	3,204	32	
65–84	79	12	1,358	14	
≥ 85	27	4	483	5	
**Sex**					
Male	331	51	4,166	42	< 0.001
Female	313	49	5,845	58	
**Major chronic conditions**					
No	421	65	6,422	64	0.530
Yes	223	35	3,589	36	
**Vaccination status in the current and three previous seasons**					
Unvaccinated	455	71	6,162	62	< 0.001
No current but any prior dose	50	8	1,049	11	
Current vaccine regardless of prior doses	139	22	2,800	28	
**Month of sample collection**					
Nov 2021	4	1	424	4	< 0.001
Dec 2021	188	29	5,416	54	
Jan 2022	100	16	2,234	22	
Feb 2022	59	9	835	8	
Mar 2022	190	30	644	6	
Apr 2022	74	12	258	3	
May 2022	29	5	200	2	

All the 190 influenza A(H3N2) strains characterised were A/Bangladesh/4005/2020(H3N2)-like (Group 3C.2a1b.2a.2); however, while in the period November–January, 89% belonged to the subgroup (iii), in February–May, 84% corresponded to the subgroup (iv).

Compared with test-negative controls, there were higher proportions of influenza cases in those younger than 25 years of age and in males. Among cases, 22% had received the current season vaccine compared with 28% of controls (Table 1).

Considering only the current season vaccination, IVE was 34% (95% CI: 13 to 49). Compared with individuals unvaccinated in the current and three previous seasons, the preventive effect observed in those vaccinated in the current season regardless of previous doses was 37% (95% CI: 16 to 52), and in those unvaccinated in the current season who had been vaccinated in any previous season was 35% (95% CI: −3 to 45). Both estimates were 40% (95% CI: 17 to 57) and 24% (95% CI: −5 to 45) in patients younger than 65 years, 10% (95% CI: −76 to 54) and 16% (95% CI: −237 to 79) in people aged 65 years and older, and 34% (95% CI: 5 to 54) and 17% (95% CI: −37 to 49) in the target population i.e. all people aged 60 years or older and those aged 6 months or over with major chronic conditions (Table 2).

**Table 2:** Effectiveness of influenza vaccination in preventing laboratory-confirmed influenza at primary care level overall, by age groups and in the target population, Navarre, Spain, 22 November 2021–22 May 2022 (n = 10,655)

Influenza vaccination status	Cases/controls n=644/10,011	Crude vaccine effectiveness % (95% CI)	Adjusted vaccine effectiveness % (95% CI)
**All patients**			
**Current season only**			
Unvaccinated	505/7,211	Ref.	Ref.
Vaccinated	139/2,800	19 (14 to 41)	34 (13 to 49)
**Current and three previous seasons**			
Unvaccinated	455/6,162	Ref.	Ref.
No current but any prior dose	50/1,049	35 (13 to 52)	35 (–3 to 45)
Current vaccine regardless of prior doses	139/2,800	33 (18 to 45)	37 (16 to 52)
**Aged ≤ 64 years**			
**Current season only**			
Unvaccinated	490/6,895	Ref.	Ref.
Vaccinated	48/1,275	47 (28 to 61)	38 (14 to 55)
**Current and three previous seasons**			
Unvaccinated	443/5,961	Ref.	Ref.
No current but any prior dose	47/934	32 (8 to 50)	24 (–5 to 45)
Current vaccine regardless of prior doses	48/1,275	49 (31 to 63)	40 (17 to 57)
**Age ≥ 65 years**			
**Current season only**			
Unvaccinated	15/316	Ref.	Ref.
Vaccinated	91/1,525	– 26 (–120 to 28)	7 (–72 to 49)
**Current and 3 previous seasons**			
Unvaccinated	12/201	Ref.	Ref.
No current but any prior dose	3/115	56 (–58 to 88)	16 (–237 to 79)
Current vaccine regardless of prior doses	91/1,525	0 (–86 to 46)	10 (–76 to 54)
**Target population^b^**			
**Current season only**			
Unvaccinated	143/2,091	Ref.	Ref.
Vaccinated	116/2,221	24 (2 to 41)	32 (3 to 52)
**Current and 3 previous seasons**			
Unvaccinated	121/1,656	Ref.	Ref.
No current but any prior dose	22/435	31 (–10 to 57)	17 (–37 to 49)
Current vaccine regardless of prior doses	116/2,221	28 (7 to 45)	34 (5 to 54)

CI: confidence interval; Ref.: reference.

^a^ Vaccine effectiveness adjusted by age groups (9–24, 25–44, 45–64, 65–84 and ≥ 85 years), sex, presence of major chronic conditions, and month.

^b^ Target population includes people aged 60 years or older and those aged 6 months or over people with major chronic conditions.

Among all ages, the vaccine effectiveness against influenza A(H3N2) was 39% (95% CI: 16 to 55) for current season vaccination regardless of prior doses and 24% (95% CI: –8 to 47) for those unvaccinated in the current season but vaccinated in any previous season. Current season IVE regardless of prior doses was 45% (95% CI: 20 to 62) in the November–January period and 23% (95% CI: –17 to 49) in February–May (p = 0.357), while people unvaccinated in the current season and vaccinated in previous seasons increased their IVE from 6% (95% CI: –43 to 38) to 42% (95% CI: 3 to 65) (Table 3).

**Table 3:** Effectiveness of influenza vaccination in preventing laboratory-confirmed influenza at primary care by calendar period and against A(H3N2) subtype, Navarre, Spain, 22 November 2021–22 May 2022 (n = 10,655 patients)

Influenza vaccination status	Cases/controls	Crude vaccine effectiveness % (95% CI)	Adjusted vaccine effectiveness^a^ % (95% CI)
**Nov 2021–Jan 2022**			
**Current season only**			
Unvaccinated	250/1,142	Ref.	Ref.
Vaccinated	102/795	41 (25 to 54)	44 (20 to 61)
**Current and three previous seasons**			
Unvaccinated	217/958	Ref.	Ref.
No current but any prior dose	33/184	21 (–18 to 47)	6 (–43 to 38)
Current vaccine regardless of prior doses	102/795	43 (27 to 56)	45 (20 to 62)
**Feb–May 2022**			
**Current season only**			
Unvaccinated	255/6,069	Ref.	Ref.
Vaccinated	37/2,005	56 (38 to 69)	17 (–26 to 45)
**Current and three previous seasons**			
Unvaccinated	238/5,204	Ref.	Ref.
No current but any prior dose	17/865	57 (29 to 74)	42 (3 to 65)
Current vaccine regardless of prior doses	37/2,005	60 (43 to 72)	23 (–17 to 49)
**A(H3N2) subtype**			
**Current season only**			
Unvaccinated	397/7,211	Ref.	Ref.
Vaccinated	116/2,800	25 (7 to 39)	36 (13 to 53)
**Current and three previous seasons**			
Unvaccinated	357/6,162	Ref.	Ref.
No current but any prior dose	40/1,049	34 (8 to 53)	24 (–8 to 47)
Current vaccine regardless of prior doses	116/2,800	28 (11 to 42)	39 (16 to 55)

CI: confidence interval; Ref.: reference.

^a^ Vaccine effectiveness adjusted by age groups (9–24, 25–44, 45–64, 65–84 and ≥ 85 years), sex, presence of major chronic conditions and month.

In the sensitivity analysis including COVID-19 positive patients in the control group, the IVE estimates were slightly lower (Supplementary Tables S1–S3).

## Discussion

Our results suggest a moderate IVE for the 2021/22 seasonal influenza vaccine of 37% against all confirmed influenza cases and of 39% influenza A(H3N2) cases, while no significant effect was observed in people aged 65 years and older at the primary care level in Navarre, Spain. Interestingly, our results also show that people vaccinated in previous seasons but not in the current season could retain some level of protection that reached statistical significance in the February–May period.

These IVE estimates were slightly higher than the in-season ones reported from Denmark and the United States [3,4]. All influenza A(H3N2) viruses characterised in the 2021/22 season in Navarre were A/Bangladesh/4005/2020-like (3C.2a1b.2a.2), which did not match the A/Cambodia/e0826360 (3C.2a1b.2a.1) vaccine component [7]. The November–January period was dominated by the subgroup (iii), while the February–May period was dominated by the subgroup (iv), as has been observed in other European countries [7]. Although it was not statistically significant, the current season IVE seemed to decline and the IVE of previous doses seemed to increase, suggesting a possible difference in affinity of both subgroups for the components of previous vaccines.

Moderate or low IVE is frequently observed in seasons with influenza A(H3N2) dominance [6,8]; furthermore, the very low influenza circulation in the 2020/21 season limited the information that supported the selection of the 2021/22 season vaccine composition [9].

Vaccines received in previous seasons may retain some preventive effect and modify the effect of the current season vaccine [6,8,10]. From the individual perspective, the total preventive benefit is the combined result of vaccinations received in the current and previous seasons. Our results suggest that strains included in vaccines of previous seasons may retain some effect against the influenza virus that circulated in the 2021/22 season. Furthermore, our IVE point estimates were slightly higher when the vaccination history was considered in the analyses.

Strengths of this study are that the comprehensive virological surveillance provided a sufficient number of cases in a season with low influenza activity and that COVID-19 confirmed cases were excluded from the control group in the main analysis to avoid possible bias [11]. To avoid biases due to vaccination information [8], the vaccination status was obtained from the regional vaccination register [5], and the study was limited to the population with stable residence in the region.

This study also has some limitations. The statistical power was limited in some analyses such as in older people or to assess changes in the IVE over time. Some level of selection bias cannot be fully excluded but it was reduced by recruiting laboratory-confirmed cases and controls in the same setting before either patient or physician were aware of laboratory results [12]. Since most COVID-19 rapid antigen test-positive patients were not tested by RT-PCR, coinfection may be underrepresented in the main analysis. This study was performed in only one region and in the special context of the COVID-19 pandemic; therefore, caution should be taken in generalising these results to other settings.

## Conclusion

Our results suggest moderate effectiveness of the 2021/22 influenza season vaccine in preventing influenza overall and, specifically, influenza A(H3N2) in outpatients, while no significant effect was observed in people older than 65 years of age and above. A possible remaining effect of previous influenza vaccine doses was seen in patients unvaccinated during the current season. Influenza vaccination provided an overall benefit even in a season with mismatch between vaccine components and the circulating influenza virus.

## Supplementary Data

280000 Supplementary Material
