# Supplementary material for: Effectiveness of influenza vaccination in preventing influenza in primary care, Navarre, Spain, 2021/22
Source: Euro Surveill. 2022 Jun 30;27(26):2200488. doi: 10.2807/1560-7917.ES.2022.27.26.2200488 (PMC9248265; doi:10.2807/1560-7917.ES.2022.27.26.2200488)
Supplement: Supplementary Material [file 22-00488_CASTILLA_influenza_Supplementary_material.pdf]

SUPPLEMENTARY MATERIAL

This supplementary material is hosted by *Eurosurveillance* as supporting information alongside the article "Effectiveness of influenza vaccination in preventing influenza in primary care, Navarre, Spain, 2021/22" on behalf of the authors who remain responsible for the accuracy and appropriateness of the content. The same standards for ethics, copyright, attributions and permissions as for the article apply. Supplements are not edited by *Eurosurveillance* and the journal is not responsible for the maintenance of any links or email addresses provided therein.

Supplementary Table S1. Characteristics of the patients with acute respiratory infection included in the test negative case-control analysis including COVID-19 confirmed cases in the control group. Navarre, Spain, 22 November 2021 to 22 May 2022 (n=16,352 patients)

	**Laboratory-confirmed influenza cases**		**Influenza negative controls**		***P* value**
	n	%	n	%	
**Total**	644	100	15,708	100	
**Age groups (years)**					< 0.001
9–24	213	33	2,352	15	
25–44	193	30	5,511	35	
45–64	132	21	5,373	34	
65–84	79	12	1,792	11	
≥85	27	4	680	4	
**Sex**					< 0.001
Male	331	51	6,371	41	
Female	313	49	9,337	59	
**Major chronic conditions**					0.575
No	421	65	10,436	66	
Yes	223	35	5,272	34	
**Vaccination status in the current and three previous seasons**					< 0.001
Unvaccinated	455	71	9,874	63	
No current but any prior dose	50	8	1,700	11	
Current vaccine regardless of prior doses	139	22	4,134	26	
**COVID-19 infection**					< 0.001
No	624	97	10,011	64	
Yes	20	3	5,697	36	
**Month of sample collection**					< 0.001
Nov 2021	4	1	534	3	
Dec 2021	188	29	7,650	49	
Jan 2022	100	16	3,580	23	
Feb 2022	59	9	1,292	8	
Mar 2022	190	30	1,831	12	
Apr 2022	74	12	459	3	
May 2022	29	5	632	2	

Supplementary Table S2. Sensitivity analysis of effectiveness of influenza vaccination in preventing laboratory-confirmed influenza at primary care level overall, by age groups and in the target population, including COVID-19 confirmed cases in the control group. Navarre, Spain, 22 November 2021 to 22 May 2022 (n=16,352 patients)

	Cases/ controls	Crude vaccine effectiveness, % (95% CI)	Adjusted vaccine effectiveness, % (95% CI)[a]
**All patients**			
**Current season only**			
Unvaccinated	505/11,574	Reference	Reference
Vaccinated	139/4,134	23 (7 to 36)	30 (10 to 46)
**Current and three previous seasons**			
Unvaccinated	455/9,874	Reference	Reference
No current but any prior dose	50/1,700	36 (14 to 53)	28 (–5 to 43)
Current vaccine regardless of prior doses	139/4,134	27 (11 to 40)	33 (13 to 48)
**Aged ≤64 years**			
**Current season only**			
Unvaccinated	490/11,069	Reference	Reference
Vaccinated	48/2,167	50 (32 to 63)	39 (16 to 55)
**Current and three previous seasons**			
Unvaccinated	443/9,545	Reference	Reference
No current but any prior dose	47/1,524	34 (10 to 51)	21 (–8 to 42)
Current vaccine regardless of prior doses	48/2,167	52 (35 to 65)	41 (19 to 57)
**Age ≥65 years**			
**Current season only**			
Unvaccinated	15/505	Reference	Reference
Vaccinated	91/1,967	–56 (–171 to 11)	–9 (–97 to 40)
**Current and three previous seasons**			
Unvaccinated	12/329	Reference	Reference
No current but any prior dose	3/176	53 (–68 to 87)	8 (–257 to 76)
Current vaccine regardless of prior doses	91/1,967	– 27 (–134 to 31)	– 7 (–106 to 44)
**Target population [b]**			
**Current season only**			
Unvaccinated	143/3,280	Reference	Reference
Vaccinated	116/3,065	13 (–11 to 32)	22 (–9 to 45)
**Current and three previous seasons**			
Unvaccinated	121/2,597	Reference	Reference
No current but any prior dose	22/683	31 (–10 to 56)	12 (–43 to 45)
Current vaccine regardless of prior doses	116/3,065	19 (–5 to 37)	24 (–7 to 46)

CI, confidence interval.
[a] Vaccine effectiveness adjusted by age groups (9-24, 25-44, 45-64, 65-84 and ≥85 years), sex, presence of major chronic conditions, and month.
[b] Target population includes people aged 60 years or older and those aged 6 months or over people with major chronic conditions.

Supplementary Table S3. Sensitivity analysis of effectiveness of influenza vaccination in preventing laboratory-confirmed influenza at primary care level by calendar period and against A(H3N2) subtype, including COVID-19 confirmed cases in the control group. Navarre, Spain, 22 November 2021 to 22 May 2022 (n=16,352 patients)

	**Cases/ controls**	**Crude vaccine effectiveness, % (95% CI)**	**Adjusted vaccine effectiveness, % (95% CI)[a]**
**November 2021 to January 2022**			
**Current season only**			
Unvaccinated	250/2,651	Reference	Reference
Vaccinated	102/1,293	17 (–6 to 34)	40 (16 to 57)
**Current and three previous seasons**			
Unvaccinated	217/2,255	Reference	Reference
No current but any prior dose	33/396	13 (–27 to 41)	–4 (–54 to 30)
Current vaccine regardless of prior doses	102/1,293	18 (–5 to 36)	40 (15 to 57)
**February 2022 to May 2022**			
**Current season only**			
Unvaccinated	255/8,923	Reference	Reference
Vaccinated	37/2,841	54 (35 to 68)	18 (–23 to 45)
**Current and three previous seasons**			
Unvaccinated	238/7,619	Reference	Reference
No current but any prior dose	17/1,304	58 (31 to 75)	43 (6 to 65)
Current vaccine regardless of prior doses	37/2,841	58 (41 to 71)	24 (–14 to 46)
**A(H3N2) subtype**			
**Current season only**			
Unvaccinated	397/11,574	Reference	Reference
Vaccinated	116/4,134	18 (–1 to 34)	32 (10 to 49)
**Current and three previous seasons**			
Unvaccinated	357/9,874	Reference	Reference
No current but any prior dose	40/1,700	35 (9 to 53)	20 (–12 to 43)
Current vaccine regardless of prior doses	116/4,134	22 (4 to 37)	35 (13 to 51)

CI: confidence interval.

[a] Vaccine effectiveness adjusted by age groups (9-24, 25-44, 45-64, 65-84 and ≥85 years), sex, presence of major chronic conditions, and month.
